# *Lichtheimia* Species Exhibit Differences in Virulence Potential

**DOI:** 10.1371/journal.pone.0040908

**Published:** 2012-07-20

**Authors:** Volker U. Schwartze, Kerstin Hoffmann, Ildikó Nyilasi, Tamás Papp, Csaba Vágvölgyi, Sybren de Hoog, Kerstin Voigt, Ilse D. Jacobsen

**Affiliations:** 1 Department of Microbial Pathogenicity Mechanisms, Leibniz Institute for Natural Product Research and Infection Biology, Hans Knöll Institute, Jena, Germany; 2 Department of Microbiology and Molecular Biology, Institute of Microbiology, University of Jena, Jena, Germany; 3 Leibniz Institute for Natural Product Research and Infection Biology, Hans Knöll Institute, Jena, Germany; 4 Department of Microbiology, Faculty of Science and Informatics, University of Szeged, Szeged, Hungary; 5 CBS-KNAW Fungal Biodiversity Centre, Utrecht, The Netherlands; Los Angeles Biomedical Research Institute, United States of America

## Abstract

Although the number of mucormycosis cases has increased during the last decades, little is known about the pathogenic potential of most mucoralean fungi. *Lichtheimia* species represent the second and third most common cause of mucormycosis in Europe and worldwide, respectively. To date only three of the five species of the genus have been found to be involved in mucormycosis, namely *L. corymbifera*, *L. ramosa* and *L. ornata.* However, it is not clear whether the clinical situation reflects differences in virulence between the species of *Lichtheimia* or whether other factors are responsible. In this study the virulence of 46 strains of all five species of *Lichtheimia* was investigated in chicken embryos. Additionally, strains of the closest-related genus *Dichotomocladium* were tested. Full virulence was restricted to the clinically relevant species while all strains of *L. hyalospora, L. sphaerocystis* and *Dichotomocladium* species were attenuated. Although virulence differences were present in the clinically relevant species, no connection between origin (environmental vs clinical) or phylogenetic position within the species was observed. Physiological studies revealed no clear connection of stress resistance and carbon source utilization with the virulence of the strains. Slower growth at 37°C might explain low virulence of *L. hyalospora*, *L. spaherocystis* and *Dichotomocladium*; however, similarly slow growing strains of *L. ornata* were fully virulent. Thus, additional factors or a complex interplay of factors determines the virulence of strains. Our data suggest that the clinical situation in fact reflects different virulence potentials in the Lichtheimiaceae.

## Introduction

Fungi of the order Mucorales are ubiquitously distributed saprophytic fungi which may cause life threatening infections in animals and humans (mucormycosis). Compared to aspergillosis and candidiasis, mucormycoses are uncommon fungal infections. However, the number of mucormycosis cases has increased during the last decades with mortality rates around 50% [1]. Main risk groups represent patients with underlying immunosuppression and diabetes but cases in immunocompetent patients have also been described [2]–[6].

*Rhizopus*, *Mucor* and *Lichtheimia* species (formerly *Absidia* or *Mycocladus*) cause 70 to 80% of all mucormycosis cases, with *Lichtheimia* species as the second and third most abundant agent in Europe and the USA, respectively [1], [3], [7], [8]. Recently, the genus *Lichtheimia* has been separated into five species, namely *L. corymbifera*, *L. ramosa*, *L. ornata*, *L. hyalospora* and *L. sphaerocystis.* To date, only the first three have been identified as causative agents in clinical cases. While *L. ornata* was only isolated twice from clinical material, *L. corymbifera* and *L. ramosa* are found more frequently [9]. However, it is not clear whether the clinical situation reflects virulence differences between the different *Lichtheimia* species or results from other factors like geographical distribution or exposure. Alastruey-Izquierdo et al. proposed that differences in thermotolerance account for clinical distribution of species [9]. However, other physiological abilities besides thermotolerance have been shown to contribute to virulence of opportunistic fungal pathogens. For example, reduced tolerance toward stresses correlates with reduced virulence in *Candida albicans* and *Aspergillus fumigatus* [10], [11], [12]. Additionally, metabolic flexibility and utilization of different nutrient sources is necessary to survive in different host niches and contributes to virulence [13], [14].

Although *Lichtheimia* species are known as pathogens since the 19^th^ century, no comprehensive study about the pathogenic potential and pathogenesis of *Lichtheimia* species exists. Comparison of the virulence potential of different strains and species requires an infection model which is inexpensive and easy to handle. Although mammalian models (especially mouse models) are still the “gold standard” for virulence studies of most pathogens, alternative infection models are increasingly used to reduce the number of mammals in experiments. Invertebrate models are widely-used to study infection and virulence [15], [16]. Although many features of the innate immunity are comparable to mammals, insect models are limited by the lack of adaptive immunity and the low optimum temperature for the insects [15], [17]–[19]. The embryonated hen egg represents an alternative infection model that has been used for infection studies on bacteria and yeasts, bridging the gap between insect and mammal infection models [20], [21]. Recently, it has been shown that embryonated eggs can be used to assay the virulence potential of *A. fumigatus* strains, with good correlation of the virulence of different strains between the chicken embryo and murine models [22].

In this study we established the chicken embryo model for *Lichtheimia* species in order to determine the virulence potential of the different *Lichtheimia* species and other thermotolerant members of the Lichtheimiaceae (*Dichotomocladium* species). Additional physiological studies were carried out to analyze if the species differ in stress resistance and nutrient acquisition and if physiological data correlate with the virulence of strains.

## Results

### Establishment of the Infection Model

**Figure 1:**
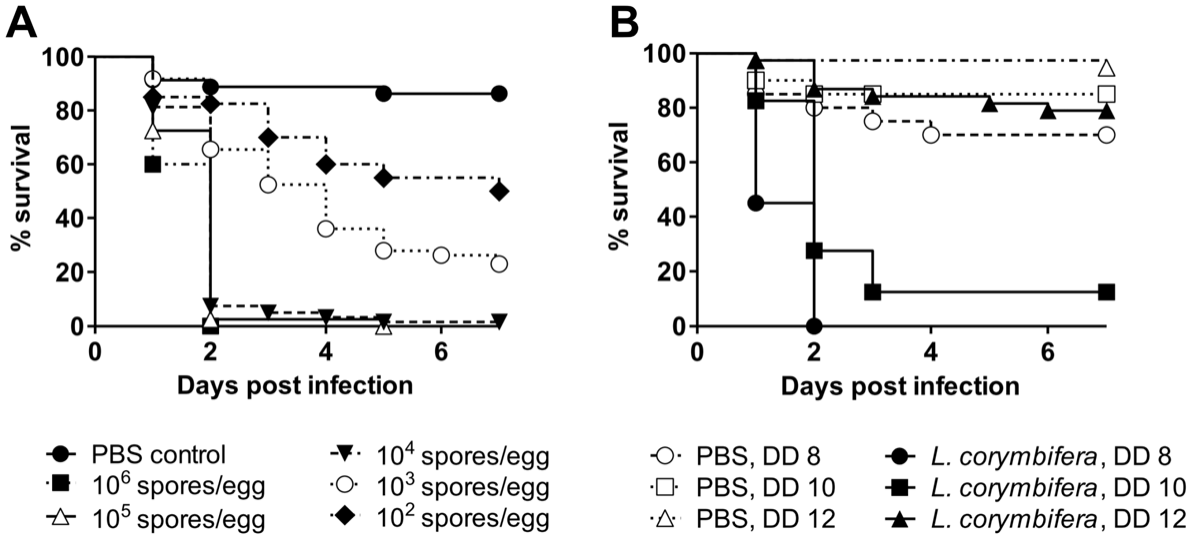
Dose- and age-dependent mortality of chicken embryos after infection with *L. corymbifera* FSU 9682. (n = 20) eggs per group, experiments were performed two (10^6^ and 10^5^ conidia per egg) to three (10^2^ to 10^4^ conidia per egg) times. Data are shown as Kaplan- Meyer curves. (A) Eggs were infected at developmental day 10 with different doses of spores as indicated. (B) Embryos were infected as indicated on developmental day (DD) 8, 10 or 12 with 10^3^ spores per egg.

To study the pathogenic potential of *Lichtheimia* species in warm-blooded hosts, the chicken embryo model was used. The neotype strain of *L. corymbifera* (FSU 9682) was used to establish the infection model. Chicken embryos were killed in a dose dependent manner by *L. corymbifera* with a LD_50_ of 100 spores (Fig. 1A). All embryos infected with 10^6^ and 10^5^ spores died within 2 to 5 days. Killing was delayed at lower doses but mortality still reached 95% to 100% (10^4^ spores) and 70% to 90% (10^3^ spores), respectively. Mortality of the embryos was dependent on the viability of the fungus since infection with spores killed by UV or thimerosal treatment resulted in nearly 100% survival of the embryos (data not shown). Experiments were repeated at least two times and displayed good reproducibility (≤5% variation between experiments).

The immune system of chicken embryos matures during development [22]. To test whether the immune status of the embryo influences the outcome of infection, embryos were infected at developmental day 8, 10 and 12 with 10^3^ spores. Infection at day 8 resulted in faster mortality compared to embryos infected at day 10. Older embryos showed lower and delayed mortality (Fig. 1B).

**Table 1:** The twelve representative *Lichtheimia* and four *Dichotomocladium* strains used throughout this study.

Species	Strain	Equivalent strain designation	Origin
*L. corymbifera* ^NT^	FSU 9682	CBS 429.75	soil
*L. corymbifera*	FSU 10164	CBS 519.71	environmental, kurone development during the manufacture of soy souce
*L. ramose*	FSU 6197	As 3.4808	environment, soil
*L. ramose*	FSU 9927	CBS 103.35	environment, fruit
*L. ramosa* ^NT^	FSU 10166	CBS 582.65	environment, seed
*L. ornata* ^T^	FSU 10165	CBS 291.66	environment, biowaste
*L. ornate*	FSU 10167	CBS 958.68	unknown
*L. hyalospora*	FSU 10160	CBS 100.28	environment, nut
*L. hyalospora*	FSU 10161	CBS 102.36	environment, stem
*L. hyalospora*	FSU 10162	CBS 518.71	environment, kurone development during the manufacture of soy souce
*L. hyalospora* ^NT^	FSU 10163	CBS 173.67	environment, fermented food
*L. sphaerocystis* ^T^	FSU 10079	CBS 420.70	unknown
*Dichotomocladium hesseltinei*	FSU 6206	CBS 164.61	environment, soil
*D. robustum*	FSU 6207	CBS 440.76	environment, dung of mouse
*D. robustum*	FSU 6208	CBS 439.76	environment, dung of mouse
*D. floridanum*	FSU 8694	IMI 349583	environment, dung of rodent

Type material is indicated with ‘T’ (type strain) or ‘NT’ (neotype strain). CBS, Centraalbureau voor Schimmelcultures Utrecht, The Netherlands; CNM-CM, Mould Collection of the Spanish National Center for Microbiology, Instituto de Salud Carlos III, Spain; IBML, Institute for Bacteriology and Mycology, Faculty of Veterinary Medicine at the University of Leipzig, Germany; FSU, Jena Microbial Resource Collection (formerly: Fungal Reference Center of the Friedrich Schiller University Jena, Germany).

### Virulence Potential of Different *Lichtheimia* Species

**Figure 2:**
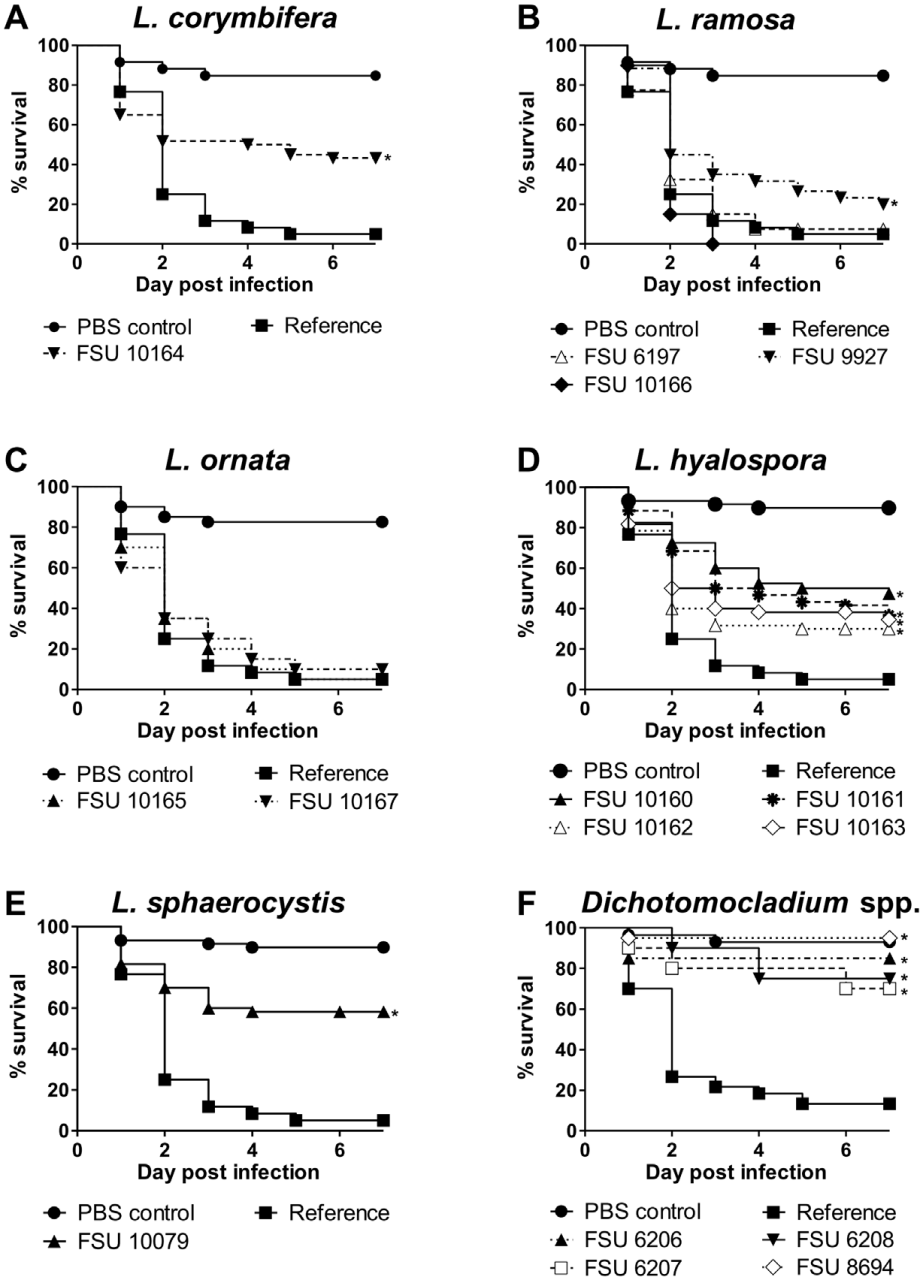
Virulence of *Lichtheimia* and *Dichotomocladium* species. 12 representative strains of *Lichtheimia* species and 4 strains of *Dichotomocladium* species were tested in embryonated eggs. Eggs were infected at developmental day 10 with 10^4^ spores per egg (n = 20 per strain). Experiments were performed at least two times. Data are shown as Kaplan- Meyer curves. Survival of embryos infected with any strain was pairwise compared to survival of embryos infected with the reference strain *L. corymbifera* FSU 9682 using the Log rank test. *: P<0.01.

Initially, the virulence potential of twelve selected representative strains, comprising type strains of all five species of *Lichtheimia* (Table 1), was determined. Survival after infection with *L. corymbifera* (FSU 9682) was used as reference value. Most of the strains from clinically relevant species *L. ramosa* and *L. ornata* showed comparable virulence to the reference (Fig. 2B–C). However, the second strain of *L. corymbifera* (FSU 10164; Fig. 2A) and one strain of *L. ramosa* (FSU 9927, Fig. 2B) showed reduced virulence. In contrast, all tested strains of *L. hyalospora* and *L. sphaerocystis* were attenuated (Fig. 2D–E), suggesting that there are differences in the pathogenic potential between *Lichtheimia* species.

To confirm this hypothesis, 34 additional strains originating from environmental, clinical and veterinary samples were tested. All additionally tested strains of *L. hyalospora* and *L. sphaerocystis* were attenuated compared to *L. corymbifera* (FSU 9682; Table 2). One quarter of the tested strains of *L. corymbifera* and *L. ramosa* showed reduced virulence. However, virulence differences did not correlate with the origin of the strains. All strains of *L. ornata* were fully virulent but strain numbers were low as only three isolates were available in the strain collections. Thus, the clinically relevant species seem to posses a higher virulence potential than *L. hyalospora* and *L. sphaerocystis*.

**Table 2:** Virulence of 46 strains of all five *Lichtheimia* species in the embryonated egg.

Origin	Number of strains	Number of attenuated strains (%)
***L. corymbifera***		
environment	4	1 (25)
animal	8	1 (12.5)
human	4	2 (50)
total	16	4 (25)
***L. ramosa***		
unknown	2	1 (50)
environment	7	2 (28.5)
animal	3	0 (0)
human	7	3 (43)
total	19	6 (31)
***L. ornata***		
unknown	1	0 (0)
environment	1	0 (0)
human	1	0 (0)
total	3	0 (0)
***L. hyalospora***		
unknown	1	1 (100)
environment	4	4 (100)
total	5	5 (100)
***L. sphaerocystis***		
unknown	1	1 (100)
environment	2	2 (100)
total	3	3 (100)

Virulence of strains from environmental, veterinarian and clinical origin was compared to the reference strain *L. corymbifera* FSU 9682 by Log rank test (P<0.01).

To determine whether other thermotolerant members of the Lichtheimiaceae also possess virulence potential, strains of the closest related genus *Dichotomocladium* were tested in the chicken embryo model. Only *D. hesseltinei*, *D. robustum* and *D. floridanum* were able to grow at 37°C and were therefore chosen for infection experiments. All strains were attenuated compared to *L. corymbifera* (FSU 9682) and mortality did not exceed 30% within 7 days (Fig. 2F).

**Figure 3:**
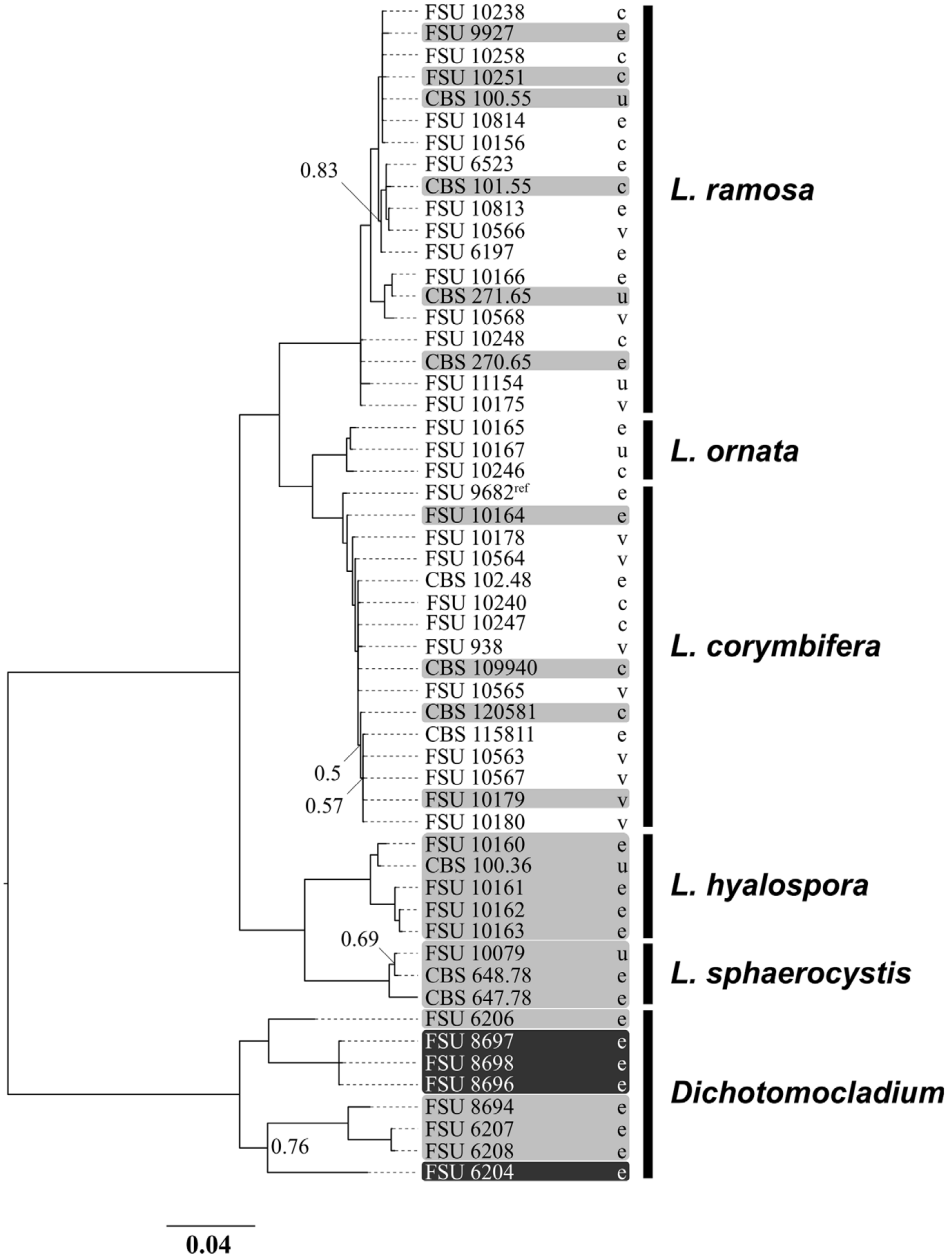
Phylogenetic tree of all used strains. Phylogenetic tree based on ITS, 28S and 18S rDNA sequences of 46 *Lichtheimia* isolates from environmental (e), veterinary (v) and clinical (c) sources. Source was not known for 6 strains (u). Strains attenuated in comparison to *L. corymbifera* FSU 9682 (ref) are highlighted in light gray. Strains which were unable to grow at 37°C (not used for infection experiments) are highlighted in dark grey. Only bootstrap values below 0.9 are indicated.

To analyze if virulent and attenuated strains represent different subgroups within the species, phylogenetic reconstruction was carried out. Phylogenetic analysis included the nuclear small subunit (18S), ITS1-5.8S rDNA-ITS2 domain and the nuclear large subunit (28S). The final alignment included 1810 nucleotides. The topology of the species reflects results from previous studies [9]. However, virulent and attenuated as well as clinical and environmental isolates were equally distributed within the species and did not cluster in subgroups (Fig. 3).

To determine whether differences in dissemination or pathological alterations account for the different virulence potential of *Lichtheimia*, eggs were infected with 10^3^ spores and eggs containing viable embryos were randomly selected. Despite significant differences in virulence between *L. corymbifera* and *L. hyalospora*, both *L. corymbifera* FSU 9682 and *L. hyalospora* FSU 10160 could be re-isolated from the CAM over seven days. None of the strains could be re-isolated from the liver indicating that no dissemination into the bloodstream occurred. Morphological examination of the CAM revealed swelling and destruction of blood vessels. However, both features were less pronounced in attenuated *L. hyalospora* compared to *L. corymbifera* (data no shown).

### Growth Rates

**Figure 4:**
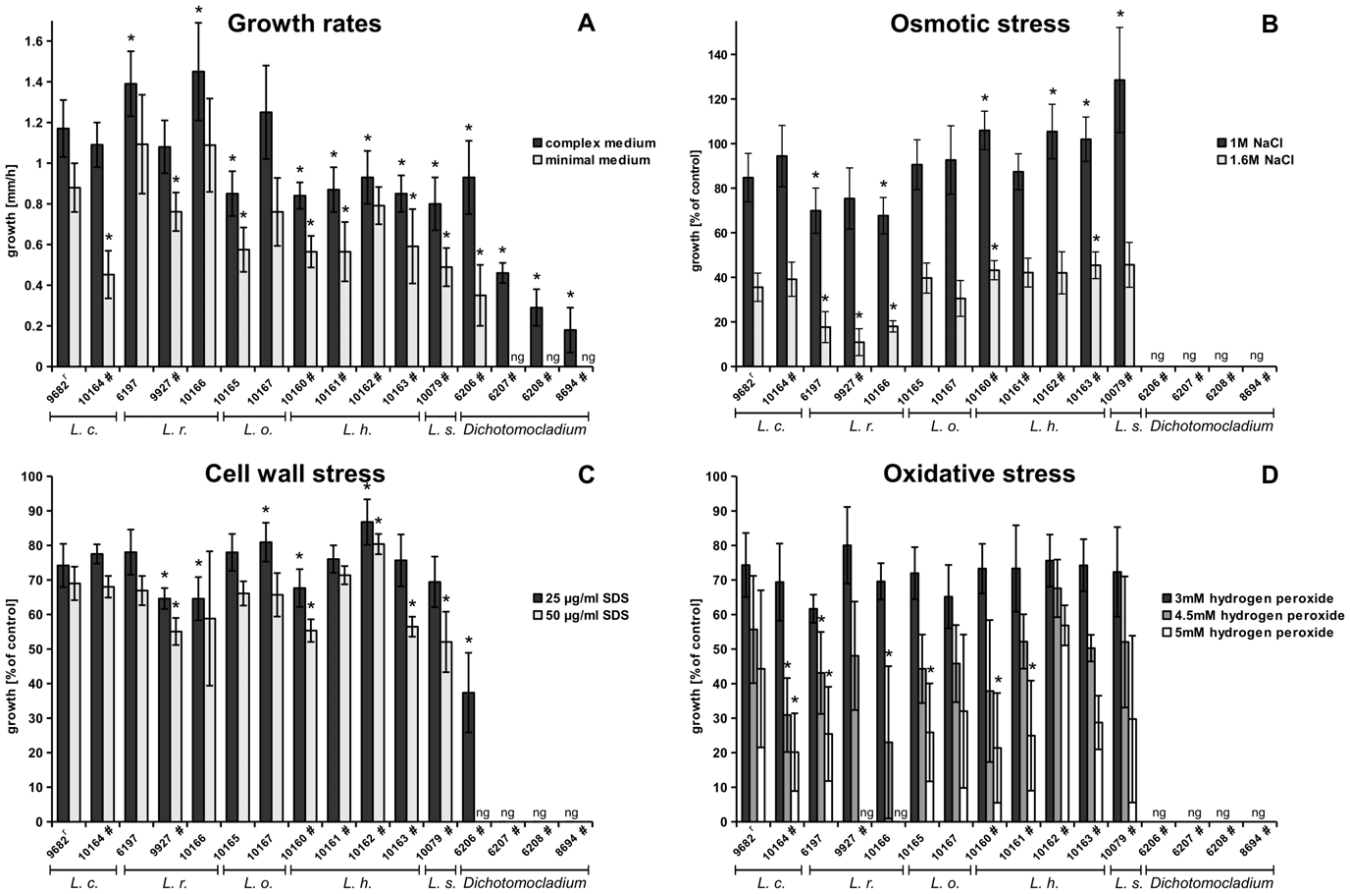
Physiological properties of the 12 representative strains of *Lichtheimia* and 4 strains of *Dichotomocladium* species. (B to D) Growth under stress is expressed as percent growth of the same strain on medium without stressor (% growth of the control). Results were compared to the reference strain *L. corymbifera* FSU 9682 (statistical significance is indicated with an asterisk; two-tailed T-test, P<0.05).

Several physiological features are essential for pathogens in order to establish infections, e.g. stress tolerance and nutrient acquisition. Furthermore, the general growth speed at host temperature may affect survival of microorganisms in the host environment. To determine whether differences in growth speed contributed to virulence differences, growth of the 12 representative strains of *Lichtheimia* and four strains of *Dichotomocladium* species was determined on both complex and minimal medium at 37°C. *L. hyalospora* and *L. sphaerocystis* grew slower on complex medium than strains of the clinically relevant species (<1 mm/h) (Fig. 4A), which is in accordance with data obtained in previous studies [9]. However, the fully virulent type strain of *L. ornata* (FSU 10165) also showed slow growth, comparable to *L. hyalospora* strains, while the attenuated *L. corymbifera* FSU 10164 grew as fast as the reference strain *L. corymbifera* FSU 9682 (1.17 mm/h). Strains of *L. ramosa* grew significantly faster than all other species (>1.3 mm/h), except the attenuated strain *L. ramosa* FSU 9927, which grew as fast as the *L. corymbifera* reference strain.

On complex medium, only three of the five species of *Dichotomocladium* were able to grow at 37°C, namely *D. hesseltinei*, *D. robustum* and *D. floridanum*. Growth of these strains was slower compared to *Lichtheimia* species (<0.6 mm/h), except of *D. hesseltinei* which grew on the level of *L. hyalospora*.

As expected, growth rates were reduced on minimal medium (<1 mm/h) compared to complex medium, except for *L. ramosa* FSU 6197 and FSU 10166 (both 1.09 mm/h) (Fig. 4A). Attenuated strains of the clinical species *L. corymbifera* (FSU 10164) and *L. ramosa* (FSU 9927) grew significantly slower than the reference strain. However, *L. ornata* FSU 10165 likewise showed significantly slower growth (comparable to *L. hyalospora*) despite being fully virulent. All strains of *L. hyalospora* and *L. sphaerocystis*, except *L. hyalospora* FSU 10162, showed reduced growth compared to the *L. corymbifera* reference strain. None of the tested *Dichotomocladium* strains were able to grow on minimal medium.

### Utilization of Carbon and Nitrogen Sources

To determine if metabolic flexibility and utilization of specific carbon sources is linked to virulence, we tested the 12 representative strains of *Lichtheimia* species and *D. hesseltinei* for growth on 66 different carbon sources (Table S2). No carbon source was exclusively used by the clinically relevant species. However, clear differences in carbon source utilization were detectable between *D. hesseltinei* and *Lichtheimia* spp. In contrast to *Lichtheimia* species, *D. hesseltinei* was unable to grow on mannite, alanine, phenylalanine and ornithine. In addition, growth of *D. hesseltinei* was inhibited by the presence of ascorbic acid, gallic acid and succinic acid.

To analyze if carbon source utilization of virulent strains showed a distinct pattern compared to attenuated strains, cluster analysis was performed using PAUP*. Therefore, the differently utilized carbon sources (n = 38) were analyzed to create a UPGMA tree. No clusters of virulent strains were detectable (Figure S1). Moreover, strains of the same species did not cluster together. However, *D. hesseltinei* was clearly separated from *Lichtheimia* species. In addition, growth on 9 different nitrogen sources was tested for the 12 representative strains of *Lichtheimia* (Table S3). No correlation between the utilization of the nitrogen sources and the virulence of the strains was detectable.

To mimic the nutrient composition within the host more accurately, we additionally determined growth on egg powder medium. All strains, except the attenuated strain *L. corymbifera* FSU 10164, grew well and formed aerial hyphae after 48 h at 37°C (data not shown).

### Stress Tolerance

Stress resistance contributes to both survival in the environment and the host. Since reduced stress resistance has been shown to reduce virulence in pathogenic fungi, e.g. *C. albicans* and *A. fumigatus* [10]–[12], the 12 representative strains of *Lichtheimia* and strains of *Dichotomocladium* species were tested for their sensitivity to osmotic, oxidative and cell wall stress, respectively. Osmotic stress was induced by either sodium chloride or sorbit. Strains of *L. corymbifera* and *L. ornata* showed similar sensitivity toward sodium chloride, whereas all strains of *L. ramosa* were reduced in their osmotolerance at least at the higher concentration (Fig. 4B). Surprisingly, strains of the attenuated species *L. hyalospora* and *L. sphaerocystis* were as or even more resistant to sodium chloride as virulent strains. Osmotic stress induced by 1.6 M sorbit had a smaller influence on the strains but revealed a similar resistance pattern as sodium chloride, except that attenuated *L. ramosa* FSU 9927 showed comparable tolerance to *L. corymbifera* strains.

Isolates of *L. ramosa* showed increased sensitivity toward cell wall stress induced by calcofluor white (CFW), while all other strains revealed resistance comparable to the reference strain *L. corymbifera* FSU 9682. Two strains of attenuated *L. hyalospora* were even more resistant than the reference strain. Additional tests with different concentrations of SDS revealed differences at the inter- and intraspecies level but showed no correlation with the virulence of the strains (Fig. 4C).

Oxidative stress was induced by different concentrations of hydrogen peroxide. As expected, growth was reduced in a dose-dependent manner. Strains of *L. hyalospora* and *L. sphaerocystis* were as tolerant or less tolerant than the *L. corymbifera* reference strain (Fig. 4D). Strains of the clinically relevant species differed in their tolerance toward oxidative stress. However, stress resistance did not correlate with the virulence potential of the respective strains. The entire species *L. ramosa* was found to be very susceptible to oxidative stress.

Strains of *Dichotomocladium* appeared to be less stress-tolerant. Species did not grow or only formed micro colonies in the presence of any of the stressors after 24 h. Only *D. hesseltinei* was able to grow on the lower dose of SDS (25 µg/ml) and on sorbit (1.6 M), but was still more susceptible than *Lichtheimia* species (Fig. 4). Thus, *Dichotomocladium* seems to be more sensitive against all tested stresses.

**Figure 5:**
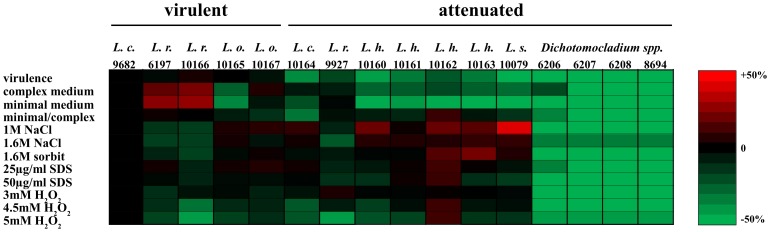
Physiology and virulence of *Lichtheimia* and *Dichotomocladium* strains. Heat map generated from virulence and physiological data of 12 representative strains of *Lichtheimia* and 4 strains of *Dichotomocladium* species. Virulence is expressed as percent mortality of chicken embryos. Growth rates are expressed as percent growth of the reference strain and stress resistance as percent growth of control without stressor. The *L. corymbifera* FSU 9682 was used as reference strain and values obtained with this strain were set as ‘0’ by subtracting the values of the reference strain from all other values.

When the physiological data and the virulence data were combined with each other (Fig. 5), no clear virulence-associated pattern across all species evolved. In *Dichotomocladium* species, slower growth and reduced stress resistance might explain the low virulence potential. However, stress resistance did not correlate with virulence of *Lichtheimia* species. Slow growth of *L. hyalospora* and *L. sphaerocystis* in comparison to *L. corymbifera* could explain the comparatively lower virulence, and attenuated strains of the clinically relevant species were reduced in growth when compared to virulent strains of the same species (*L. ramosa* FSU 9927) or showed defects in growth on minimal medium (*L. corymbifera* FSU 10164). However, slowly growing strains of *L. ornata* were found to be fully virulent.

### Revision of the Taxonomic Designation of *Lichtheimia* Strains

**Figure 6:**
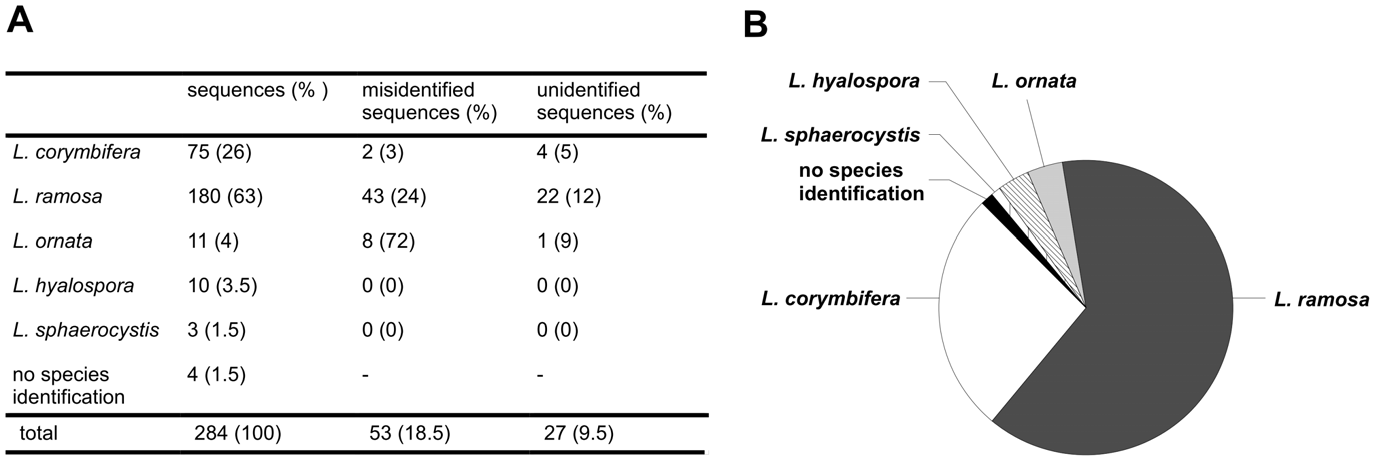
Species designation of all ITS sequences of *Lichtheimia* in GenBank. Number of sequences and percent of misidentified sequences (A). Chart pie of proportion of sequences of the different species of *Lichtheimia* (B).

Although *L. ornata* was found to be fully virulent in our infection model, it was isolated only two times from clinical material [9]. Since species of *Lichtheimia* were regarded as synonyms for a long time [9], [23] and fungal specimens are mainly identified by comparison of the ITS sequence against public databases (e.g. GenBank), isolates of *L. ornata* may be misapplied. To test this hypothesis, we analyzed all sequences deposited in GenBank which were identified as *Lichtheimia* species (or their synonyms *Mycocladus* or *Absidia*). Additionally, we did a BLAST search with sequences of the type strains from all five *Lichtheimia* species to identify misapplied or unidentified isolates. In total we found 284 sequences in GenBank (as of November 11^th^ 2011). Sequences were assigned to the species by Neighbor Joining analysis. *L. ramosa* and *L. corymbifera* represented the most common species, while *L. ornata* represented only 4% of all sequences (Fig. 6). However, only three of the eleven sequences identified as corresponding to *L. ornata* were properly annotated in GenBank. The other sequences were deposited as *L. corymbifera* (6), *L. ramosa* (1) or *Lichtheimia* sp. (1).

In contrast, *L. ramosa* and *L. corymbifera* represented 63% and 26%, respectively, of all sequences deposited in GenBank. Most sequences were correctly designated to *L. corymbifera,* while almost one quarter of the *L. ramosa* sequences were ascribed to *L. corymbifera*, the former synonym of *L. ramosa*. All sequences from *L. hyalospora* and *L. sphaerocystis* were correctly applied.

Surprisingly, only two unidentified sequences of uncultured environmental samples were found to belong to *Lichtheimia* species.

## Discussion

Despite the increasing acknowledgment as causative agents of disease [1], [7], [8], little is known about the pathogenesis and pathogenic potential of mucoralean species, especially members of the genus *Lichtheimia*. In an attempt to determine the virulence potential of different *Lichtheimia* species, we used chicken embryos as an inexpensive and easy-to-handle infection model for mucormycosis. The embryos were susceptible to infection with *Lichtheimia* species in a dose and age-dependent manner with a LD_50_ of 100 spores. Compared to other fungal pathogens, virulence of *L. corymbifera* in the embryonated egg model was higher than *C. albicans* (LD_50_ 10^5^ cfu per egg) but lower than *A. fumigatus* (90% killing at 100 spores per egg) [22], [24].

Tissue invasion and destruction are hallmarks for mucormycosis in human patients [25], [26]. In a recent study, *R. arrhizus* (syn. *R. oryzae*) *wa*s shown to be able to invade endothelial cells with their subsequent damage as surveyed by *in vitro* assays [27]. Additionally, it was also shown that invasion depends on the glucose-regulated endothelial receptor GRP78 and blocking of the interaction results in strongly decreased mortality in mice [28]. Thus, interaction with blood vessels seems to play a crucial role in the pathogenesis of mucormycosis. We likewise observed tissue invasion and destruction of blood vessels in chicken embryos and found that destruction of blood vessels was less pronounced in attenuated *L. hyalospora* compared to virulent *L. corymbifera*, suggesting that similar pathogenesis mechanisms contribute to virulence in the embryonated egg model. Although destruction of blood vessels took place early during infection, no dissemination of the fungus into the liver was observed.

Only *L. corymbifera*, *L. ramosa* and *L. ornata* are recognized as clinically relevant species. Our finding that these three species had a higher virulence potential than *L. hyalospora* and *L. sphaerocystis* suggests that this distribution is attributable to differences in virulence rather than lower frequency of infection due to lower abundance in the environment. Within the clinically relevant species, *L. ornata* was isolated only twice from clinical material [9], though our results suggest a virulence potential comparable to the other clinical species. Since only three well characterized stains of *L. ornata* were available in our culture collections, it could be assumed that *L. ornata* is less abundant than the other clinical species. This is supported by our analysis of the *Lichtheimia* sequences obtained from GenBank, where *L. ornata* represented only 4% of all sequences, while *L. corymbifera* and *L. ramosa* encompassed almost 90%. Thus, the lack of *L. ornata* infections might in fact result from the lower abundance of this species in the environment. However, ecological studies on the distribution and abundance of *Lichtheimia* species have not been conducted so far and it can not be excluded that other factors contribute to the lower frequency of *L. ornata* infections.

It has been suggested that environmental and clinical strains of fungal pathogens might differ in their virulence potential. However, conflicting data has been published for *A. fumigatus* and *Cryptococcus neoformans*, as only some but not all studies found clinical strains to be more virulent [29]–[32]. Although we observed significant variation of the virulence potential within clinically relevant *Lichtheimia* species, these differences did not depend on the origin (environmental *versus* clinical and geographical region) of the strains. Furthermore, clinical and environmental as well as virulent and attenuated strains were equally distributed within the species in phylogenetic reconstruction, indicating that virulent strains represent no specific subgroups within the species. Thus, it appears likely that most strains of *L. corymbifera*, *L. ramosa* and *L. ornata* (regardless of environmental or clinical origin) exhibit the potential to cause infections in susceptible hosts.

During mucormycosis, fungi grow within the host tissue. Thus, general physiological attributes which contribute to survival and replication with the host environment are likely to contribute to the virulence potential. Within virulent species, attenuated strains showed slower growth or growth defects on minimal media compared to virulent strains of the same species, suggesting a correlation between growth and virulence. This is in accordance with the results for *A. fumigatus*, where strains with reduced growth at 37°C were significantly less virulent [33]. Similarly, the slower growth rate of *L. hyalospora*, *L. sphaerocystis* and *Dichotomocladium* species on complex and minimal media correlated with lower virulence. In contrast, comparatively slow growing *L. ornata* strains were fully virulent, suggesting that growth alone does not sufficiently explain differences in virulence.

The utilization of macronutrients, e.g. carbon sources, in the host affects growth *in vivo* and might thus contribute to virulence. Although we observed differences in carbon source utilization, the patterns did not correspond with the virulence potential of strains. However, within the host, combinations of nutrients are available and various utilization patterns may favor or restrict growth, respectively. Furthermore, nutrients within the host are most likely available as complex molecules, e. g. proteins, rather than free amino acids. With the exception of *L. corymbifera* FSU 10164, all strains grew well on egg powder medium, suggesting that all strains could sufficiently utilize host nutrients for growth. Thus, it appears unlikely that differences in virulence between *Lichtheimia* species are due to varying ability to assimilate macronutrients in the host.

Chicken embryos have been shown to mount an immune response towards fungal and bacterial pathogens, including recruitment of phagocytes to the site of infection [22], [24], [34], thus creating a hostile environment for microorganisms. Resistance against stressors thereby influences microbial survival and virulence. Surprisingly, *L. hyalospora* and *L. sphaerocystis* were at least as stress resistant as the virulent strains of *L. corymbifera*, *L. ramosa* and *L. ornata*, suggesting that virulence differences are not mediated by resistance to the tested stresses. In comparison to the *Lichtheimia* species, *Dichotomocladium* species showed strongly reduced growth at 37°C and impaired resistance against various stresses, which likely leads to decreased survival in the infection models and thus explains the low virulence.

In summary, our data show that, in the alternative infection model used in this study, *L. corymbifera*, *L. ramosa* and *L. ornata* have a higher virulence potential than *L. hyalospora* and *L. sphaerocystis*, which correlates with the recognition of *L. corymbifera*, *L. ramosa* and *L. ornata* as clinically relevant species. However, we were unable to identify physiological traits which are clearly correlated with virulence, implicating that virulence of *Lichtheimia* species either results from a complex interplay of physiological features or that specific factors which were not analyzed in this study significantly contribute to the virulence potential. Importantly, if *Lichtheimia* species are isolated from clinical specimens, species identification by sequencing but not classical microbiological analyses might provide information on the putative virulence potential of the isolate.

## Materials and Methods

### Ethics Statement

All experiments were performed in compliance with the European and German animal protection law. According to this, no specific approval is needed for work performed in avian embryos before the time of hatching. The experimental protocols were reviewed and approved in regard to ethical and welfare issues by the responsible animal welfare officer. Experiments were terminated latest on developmental day 18, three days before hatching, by chilling the eggs on ice for 30–60 min.

### Fungal Strains and Cultivation

A total of 54 strains comprising 46 strains of *Lichtheimia* spp. and 8 strains of *Dichotomocladium* spp. were used for this study (Table S1). The strains are available from the Centraalbureau voor Schimmelcultures Utrecht, The Netherlands (CBS); the mold collection of the Spanish National Centre for Microbiology, Instituto de Salud Carlos III (CNM-CM) and the Jena Microbial Resource Collection (JMRC). Strains were cultivated at 37°C or room temperature as indicated on modified SUP medium [35]: 55 mM glucose, 30 mM potassium dihydrogen phosphate, 20 mM ammonium chloride, 5 mM di-potassium hydrogen phosphate, 1 mM magnesium sulphate and 0.5% yeast extract. Spores were washed off fully grown cultures with sterile PBS (137 mM NaCl, 10 mM Na_2_HPO_4_, 2.7 mM KCl, 1.76 mM KH_2_PO_4_, pH7.4), counted microscopically in a Thoma counting chamber and diluted to the indicated concentrations with PBS.

### Chicken Embryo Model

Chicken embryos were infected via the chorio-allantoic membrane (CAM) as described previously [20], [22] with 100 µl spore suspension (spore concentration indicated in results). Unless stated otherwise, embryos were infected at developmental day (DD) 10. Twenty eggs were used per group and experiment. Survival was determined daily by candling.

### Extraction of Genomic DNA, Sequencing and Phylogenetic Reconstruction

Genomic DNA was purified according to a modified method of Cenis (1993) as described previously [36], [37]. For phylogenetic analysis, sequences of the internal transcribed spacer (ITS) region spanning ITS1-5.8S-ITS2, the D1/D2 region of the nuclear large subunit (LSU) and nuclear small subunit (SSU) were analyzed. PCR was performed using ITS1 and ITS4 (ITS1∶5′-TCCGTAGGTGAACCTGCGG-3′, ITS4∶5′-TCCTCCGCTTATTGATATGC-3′; [38]), NL1 and NL4 (NL1∶5′-GCATATCAATAAGCGGAGGAAAAG-3′, NL4∶5′-GGTCCGTGTTTCAAGACGG-3′, [39]) and NS1 and NS41 (NS1∶5′-GTAGTCATATGCTTGTCTC-3′, [38]; NS41∶5′-CCCGTGTTGAGTCAAATTA-3′, [40]). 50–100 ng genomic DNA were used as template in standard PCR.

PCR fragments were purified according to the method of Vogelstein and Gillespie (1979) [41]. Sequencing was performed in 96 well plates on an Applied Biosystems 3730xL DNA Analyzer (ABI, Carlsbad) according to the manufacturer’s instructions.

Sequences were aligned by ClustalW [42] using BioEdit version 7.0.5.3 [43]. The combined alignment consisted of nucleotide sequences of LSU, SSU and ITS with 406, 835 and 519 characters, respectively. The online version of MrBayes on TG on the CIPRES portal (www.phylo.org) was used for phylogenetic reconstruction. Two independent runs with 5,000,000 generations were used and trees were sampled every 2,000 generations. Burn-in was set to 0.25.

### Analysis of GenBank Sequences

All ITS sequences deposited in GenBank as *Lichtheimia* or one of their synonyms (*Mycocladus*, *Absidia*) were taken into account. Additionally, sequences of the type strains of the five species were used to perform a BLAST analysis against all non-*Lichtheimia* sequences to identify potential mis- or unidentified sequences. A total of 284 sequences were aligned using the online version of MAFFT [44], and Neighbour Joining analysis [45] as implemented in the MAFFT online version (http://mafft.cbrc.jp/alignment/software/) was performed with 1,000 bootstrap replications using *Mucor circinelloides* CBS 195.68 (Acc. No.: JF723597.1) as outgroup.

### Growth Rates

Five µl spore suspension with 10^5^ spores per ml were placed in the middle of a petri dish containing either SUP medium or minimal medium (SUP medium without yeast extract). Plates were incubated at 37°C for two days and colony diameter were measured daily. Growth experiments were performed three times in duplicate.

### Carbon and Nitrogen Source Utilization

For inoculation of carbon source utilization experiments, 20 µl spore suspensions (10^6^ spores per ml) were dropped onto the surface of MM (0.5% (NH_4_)_2_SO_4_, 0.1% KH_2_PO_4_, 0.05% MgSO_4_ x 7H_2_O) agar plates containing a single carbon source at a concentration of 0.2% (w/v). Incubation was performed at 28°C and growth characteristics were analyzed after 4, 6 and 10 days. Cluster analysis (UPGMA [46]) of differently utilized carbon sources was performed using PAUP* version 4.0 beta [47]. Distance option “mean number of pairwise character differences” was used. Growth data were categorized as growth, slight growth, no growth, slight inhibition and inhibition. For the test of growth on different nitrogen sources strains were starved on minimal medium without nitrogen source for 7 days at 37°C. Small agar blocks were transferred to plates containing 0.5% Yeast Nutrient Broth, 1% glucose and a single nitrogen source at a concentration of 0.2% for tryptophane and asparagine and 0.5% for all other nitrogen sources. Plates were incubated at 37°C for 5 days. For growth tests on egg powder medium (5% egg powder [Fluca], 30 mM potassium dihydrogen phosphate, 5 mM di-potassium hydrogen phosphate, 1 mM magnesium sulphate) plates were inoculated with 5 µl of a spore suspension with 10^5^ spores per ml. Plates were incubated at 37°C and analyzed after 2 days.

### Stress Resistance

SUP medium was supplemented with either sodium chloride, sorbit, sodium dodecyl sulphate (SDS), calcofluor (Fluorescence brightener 28, Sigma Aldrich; CFW) or hydrogen peroxide at indicated concentrations. Plates were inoculated as described above and incubated at 37°C for 24 h. Experiments were performed three times in triplicate. Data were statistically analyzed by unpaired t-test using GraphPad Prism version 5.00 for Windows (GraphPad Software, San Diego, CA).

## Supporting Information

Figure S1 **Pattern of carbon source assimilation of Lichtheimia species.** Dendrogram based on the different usage of 38 carbon sources by 12 representative strains of *Lichtheimia* species and *D. hesseltinei*. UPGMA tree was generated using PAUP*. Growth data were categorized as growth, slight growth, no growth, slight inhibition and inhibition.
(TIF)

Table S1 **List of all strains (54) used in this study.** Representative strains also listed in Table 1 are printed in bold. Type material is indicated with ‘T’ (type strain) or ‘NT’ (neotype strain). CBS, Centraalbureau voor Schimmelcultures Utrecht, The Netherlands; CNM-CM, Mould Collection of the Spanish National Center for Microbiology, Instituto de Salud Carlos III, Spain; IBML, Institute for Bacteriology and Mycology, Faculty of Veterinary Medicine at the University of Leipzig, Germany; FSU, Jena Microbial Resource Collection (formerly: Fungal Reference Centre of the Friedrich Schiller University Jena, Germany).
(DOC)

Table S2 **Utilization of 66 carbon sources by 12 representative strains of** ***Lichtheimia*** **species and** ***D. hesseltinei*** **.** No growth is indicated as ‘0’, growth inhibition as ‘-’ and growth as ‘+’ compared to medium without a carbon source.
(DOC)

Table S3 **Utilization of different nitrogen sources by 12 representative strains of** ***Lichtheimia*** **species.** No Growth is indicated as ‘0’, inhibition as ‘-’ and growth as ‘+’.
(DOCX)
