# Supplementary material for: Lichtheimia Species Exhibit Differences in Virulence Potential
Source: PLoS One. 2012 Jul 20;7(7):e40908. doi: 10.1371/journal.pone.0040908 (PMC3401187; doi:10.1371/journal.pone.0040908)
Supplement: Table S1 — List of all strains (54) used in this study. Representative strains also listed in Table 1 are printed in bold. Type material is indicated with ‘T’ (type strain) or ‘NT’ (neotype strain). CBS, Centraalbureau voor Schimmelcultures Utrecht, The Netherlands; CNM-CM, Mould Collection of the Spanish National Center for Microbiology, Instituto de Salud Carlos III, Spain; IBML, Institute for Bacteriology and Mycology, Faculty of Veterinary Medicine at the University of Leipzig, Germany; FSU, Jena Microbial Resource Collection (formerly: Fungal Reference Centre of the Friedrich Schiller University Jena, Germany). (DOC) [file pone.0040908.s002.doc]

**Table S1. List of all strains (54) used in this study.** Representative strains also listed in Table 1 are printed in bold. Type material is indicated with 'T' (type strain) or 'NT' (neotype strain). CBS, Centraalbureau voor Schimmelcultures Utrecht, The Netherlands; CNM-CM, Mould Collection of the Spanish National Center for Microbiology, Instituto de Salud Carlos III, Spain; IBML, Institute for Bacteriology and Mycology, Faculty of Veterinary Medicine at the University of Leipzig, Germany; FSU, Jena Microbial Resource Collection (formerly: Fungal Reference Centre of the Friedrich Schiller University Jena, Germany).

| **Species** | **Strain** | **Equivalent strain designation** | **Origin** | **ITS GenBank accession no.** | **NL GenBank accession no.** | **NS GenBank accession no.** |
| --- | --- | --- | --- | --- | --- | --- |
| *Lichtheimia corymbifera* | FSU 938 | CBS 100.31 | bovine fetus | AY944895.1 | FJ719429 | AF113407 |
| *L. corymbifera* ^NT^ | FSU 9682 | CBS 429.75 | soil | GQ342878 | GQ342903 | JQ775437 |
| *L. corymbifera* | CBS 109940 | FSU 10061 | human, finger tissue, Norway | GQ342881.1 | GQ342917 | JQ775443 |
| *L. corymbifera* | CBS 120581 | FSU 10074 | human, bronchia, France | GQ342883.1 | GQ342948 | JQ775444 |
| *L. corymbifera* | FSU 10164 | CBS 519.71 | environmental, kurone development during the manufacture of soy souce | GQ342889 | GQ342904 | JQ775438 |
| *L. corymbifera* | FSU 10178 | IBML 4 – M 10012 | cattle, gut | JQ775395 | JQ775488 | JQ775459 |
| *L. corymbifera* | FSU 10179 | IBML 5 – M 10005 | horse, gut | JQ775396 | JQ775489 | JQ775460 |
| *L. corymbifera* | FSU 10180 | IBML 6 – D 10005 | horse, gut | JQ775397 | JQ775490 | JQ775461 |
| *L. corymbifera* | FSU 10240 | CNM – CM 3415 | human, ear swab | HM104196 | JQ775475 | JQ775439 |
| *L. corymbifera* | FSU 10247 | CNM – CM 5039 | human, peritoneal drainage | HM104200 | JQ775477 | JQ775441 |
| *L. corymbifera* | FSU 10563 | P. O. 623 | stork, lung | JQ775398 | JQ775480 | JQ775451 |
| *L. corymbifera* | FSU 10564 | P. O. 829 | stork, lung | JQ775399 | JQ775481 | JQ775452 |
| *L. corymbifera* | FSU 10565 | P. O. 612 B | stork, lung | JQ775407 | JQ775479 | JQ775450 |
| *L. corymbifera* | FSU 10567 | P. O. 909 B | stork, lung | JQ775402 | JQ775484 | JQ775455 |
| *L. corymbifera* | CBS 102.48 | FSU 10806 | environment, mouldy shoe | GQ342888 | GQ342910 | JQ775418 |
| *L. corymbifera* | CBS 115811 | FSU 10807 | environment, indoor air | GQ342887 | GQ342932 | JQ775417 |
| *L. ramosa* | CBS 100.55 | FSU 748 | unknown | AB305110 | GQ342938 | JQ775442 |
| *L. ramosa* | CBS 270.65 | FSU 787 | environment | AY944897.1 | FJ719445 | JQ775428 |
| *L. ramosa* | CBS 271.65 | FSU 788 | unknown | AY944897.1 | GQ342937 | JQ775429 |
| *L. ramosa* | FSU 6197 | As 3.4808 | environment, soil | GQ342867 | GQ342955 | EU826361.1 |
| *L. ramosa* | FSU 6523 | - | environment | GQ221211 | JQ775474 | JQ775422 |
| *L. ramosa* | FSU 9927 | CBS 103.35 | environment, fruit | GQ342847 | GQ342908 | JQ775424 |
| *L. ramosa* | CBS 101.55 | FSU 10000 | human, cornea | GQ342865 | GQ342947 | JQ775445 |
| *L. ramosa* | FSU 10156 | - | human, stool | JQ775405 | JQ775487 | JQ775458 |
| *L. ramosa* ^NT^ | FSU 10166 | CBS 582.65 | environment, seed | GQ342874 | GQ342909 | JQ775425 |
| *L. ramosa* | FSU 10175 | IBML 1 – D 10007 | cattle, gut | JQ775404 | JQ775486 | JQ775457 |
| *L. ramosa* | FSU 10238 | CNM – CM 3013 | human, wound | HM104194 | JQ775472 | JQ775419 |
| *L. ramosa* | FSU 11154 | - | unknown | JQ775408 | JQ775476 | JQ775440 |
| *L. ramosa* | FSU 10248 | CNM – CM 5111 | human, sputum | GQ342871 | JQ775473 | JQ775420 |
| *L. ramosa* | FSU 10251 | CNM – CM 5399 | human, bronchioaspirate | HM104211 | JQ775471 | JQ775427 |
| *L. ramosa* | FSU 10258 | CNM – CM 5396 | human, bronchoalveolar lavage | HM104208 | JQ775470 | JQ775426 |
| *L. ramosa* | FSU 10566 | P. O. 905 A | stork, lung | JQ775400 | JQ775482 | JQ775453 |
| *L. ramosa* | FSU 10568 | P. O. 909 A | stork, lung | JQ775401 | JQ775483 | JQ775454 |
| *L. ramosa* | FSU 10813 | **-** | environment, straw | JQ775406 | JQ775478 | JQ775449 |
| *L. ramosa* | FSU 10814 | - | environment, biowaste | JQ775403 | JQ775485 | JQ775456 |
| *L. ornata* ^T^ | FSU 10165 | CBS 291.66 | environment, dung of bird | GQ342891 | GQ342946 | JQ775430 |
| *L. ornata* | FSU 10167 | CBS 958.68 | unknown | GQ342890 | GQ342936 | JQ775431 |
| *L. ornata* | FSU 10246 | CNM -CM 4978 | human, wound | GQ342892 | GQ342952.1 | JQ775432 |
| *L. hyalospora* | FSU 10160 | CBS 100.28 | environment, nut | GQ342896 | GQ342902 | JQ775433 |
| *L. hyalospora* | FSU 10161 | CBS 102.36 | environment, stem | GQ342895 | GQ342907 | JQ775434 |
| *L. hyalospora* | FSU 10162 | CBS 518.71 | environment, kurone development during the manufacture of soy souce | GQ342894 | GQ342944 | JQ775435 |
| *L. hyalospora* ^NT^ | FSU 10163 | CBS 173.67 | environment, fermented food | GQ342893 | GQ342905 | JQ775436 |
| *L. hyalospora* | CBS 100.36 | FSU 10808 | unknown | GQ342898 | GQ342943 | JQ775448 |
| *L. sphaerocystis* ^T^ | FSU 10079 | CBS 420.70 | unknown | GQ342900 | GQ342900 | JQ775421 |
| *L. sphaerocystis* | CBS 647.78 | FSU 10638 | environment, dung of mouse | GQ342899 | GQ342911 | JQ775446 |
| *L. sphaerocystis* | CBS 648.78 | FSU 10640 | environment, soil | GQ342901 | GQ342916 | JQ775447 |
| *Dichotomocladium hesseltinei* | FSU 6206 | CBS 164.61 | environment, soil | JQ775411 | JQ775493 | JQ775464 |
| *D. robustum* | FSU 6207 | CBS 440.76 | environment, dung of mouse | JQ775412 | JQ775494 | JQ775465 |
| *D. robustum* | FSU 6208 | CBS 439.76 | environment, dung of mouse | JQ775413 | JQ775495 | JQ775466 |
| *D. floridanum* | FSU 8694 | IMI 349583 | environment, dung of rodent | JQ775409 | JQ775491 | JQ775462 |
| *D. elegans* | FSU 6204 | CBS 714.74 | environment, dung of mouse | JQ775410 | JQ775492 | JQ775463 |
| *D. sphaerosporum* | FSU 8696 | IMI 338704 | unknown | JQ775416 | JQ775498 | JQ775469 |
| *D. sphaerosporum* | FSU 8697 | IMI 236324 | unknown | JQ775414 | JQ775496 | JQ775467 |
| *D. sphaerosporum* | FSU 8698 | IMI 236226 | unknown | JQ775415 | JQ775497 | JQ775468 |
