# Supplementary material for: Lichtheimia Species Exhibit Differences in Virulence Potential
Source: PLoS One. 2012 Jul 20;7(7):e40908. doi: 10.1371/journal.pone.0040908 (PMC3401187; doi:10.1371/journal.pone.0040908)
Supplement: Table S2 — Utilization of 66 carbon sources by 12 representative strains of Lichtheimia species and D. hesseltinei . No growth is indicated as ‘0’, growth inhibition as ‘-’ and growth as ‘+’ compared to medium without a carbon source. (DOC) [file pone.0040908.s003.doc]

**Table S2. Utilization of 66 carbon sources by 12 representative strains of *Lichtheimia* species and *D. hesseltinei*.** No growth is indicated as '0', growth inhibition as '-' and growth as '+' compared to medium without a carbon source.

|  | ***L. corymbifera*** | | ***L. ramosa*** | | | ***L. ornata*** | | ***L. hyalospora*** | | | | ***L. sphaerocystis*** | ***D. hesseltinei*** |
| --- | --- | --- | --- | --- | --- | --- | --- | --- | --- | --- | --- | --- | --- |
|  | **FSU 9682** | **FSU 10164** | **FSU 6197** | **FSU 9927** | **FSU 10166** | **FSU 10165** | **FSU 10167** | **FSU 10160** | **FSU 10161** | **FSU 10162** | **FSU 10163** | **FSU**  **10079** | **FSU**  **6206** |
| **D-glucose** | +++ | +++ | +++ | +++ | +++ | +++ | +++ | +++ | +++ | +++ | +++ | +++ | ++ |
| **sucrose** | 0 | 0 | 0 | 0 | 0 | 0 | 0 | 0 | 0 | ++ | ++ | 0 | 0 |
| **D-lyxose** | 0/+ | + | + | + | + | + | ++ | 0/- | 0/- | + | + | +++ | 0 |
| **D-xylose** | ++ | +++ | ++ | +++ | +++ | +++ | ++++ | +++ | ++++ | ++ | ++ | +++ | +++ |
| **D-fructose** | ++ | +++ | +++ | ++ | +++ | ++ | +++ | ++ | +++ | +++ | ++ | +++ | +++ |
| **D-galactose** | +++ | +++ | +++ | +++ | +++ | ++ | +++ | ++ | +++ | ++ | ++ | +++ | ++ |
| **D-mannose** | +++ | +++ | +++ | ++ | +++ | ++ | +++ | +++ | +++ | +++ | ++ | +++ | ++ |
| **L-arabinose** | +++ | +++ | +++ | ++ | ++++ | ++ | +++ | +++ | +++ | ++ | +++ | +++ | +++ |
| **maltose** | ++ | ++ | +++ | +++ | +++ | + | ++ | ++ | +++ | ++ | +++ | +++ | ++ |
| **myo-inositol** | 0 | 0 | + | 0/+ | + | 0 | 0 | 0 | 0 | 0 | 0 | 0 | 0 |
| **cellobiose** | +++ | ++ | +++ | ++ | +++ | ++ | ++ | ++ | +++ | ++ | ++ | ++ | ++ |
| **raffinose** | 0/+ | + | ++ | 0/+ | + | + | ++ | 0/+ | 0/+ | + | +++ | + | + |
| **starch** | ++ | + | +++ | + | ++++ | + | +++ | + | ++ | ++ | ++ | ++ | ++ |
| **sorbit** | +++ | ++ | +++ | +++ | +++ | + | ++ | ++ | ++ | + | + | +++ | 0 |
| **xylitol** | + | 0/+ | ++ | + | +++ | +++ | ++ | ++ | +++ | ++ | +++ | +++ | 0 |
| **Gln** | + | ++ | + | + | + | + | + | + | + | 0/+ | + | ++ | 0/+ |
| **Pro** | ++ | + | ++ | +++ | ++ | + | + | ++ | 0/+ | 0/+ | 0/+ | ++ | 0 |
| **Asn** | ++ | ++ | ++ | 0/+ | + | + | ++ | + | + | 0/+ | 0 | ++ | 0/+ |
| **Glu** | +++ | ++ | +++ | ++ | ++ | ++ | +++ | +++ | ++ | ++ | +++ | +++ | 0/+ |
| **ornithine** | +++ | +++ | ++ | ++ | ++ | ++ | +++ | +++ | +++ | + | ++ | +++ | 0 |
| **Glycerol-l-monohydrate** | 0 | 0 | 0 | 0 | 0 | 0 | 0 | 0 | 0 | 0 | 0 | 0 | 0 |
| **succinic acid** | + | ++ | ++ | + | +++ | ++ | ++ | + | + | + | + | ++ | - |
| **gluconic acid** | 0/+ | 0 | + | 0/+ | + | + | 0/+ | 0 | 0 | 0 | 0/+ | + | 0 |
| **L-sorbose** | 0 | 0/+ | 0/+ | 0 | 0 | 0 | 0 | 0 | 0 | 0/- | 0 | 0 | 0 |
| **L-rhamnose** | 0 | 0 | 0 | 0 | 0 | 0 | 0 | 0 | 0 | 0 | 0 | 0 | 0 |
| **lactose** | ++ | ++ | +++ | + | ++++ | + | +++ | ++ | +++ | ++ | ++ | +++ | ++ |
| **xylan** | 0/+ | 0 | 0/+ | 0 | 0 | 0 | 0 | 0 | 0 | 0 | 0 | 0/+ | 0/+ |
| **dextran** | 0 | 0 | 0 | 0 | 0 | 0 | 0 | 0 | 0 | 0 | 0 | 0 | 0/+ |
| **inulin** | 0 | 0 | 0 | 0 | 0 | 0 | 0 | 0 | 0 | 0 | 0 | 0 | 0 |
| **L-maleic acid** | + | - | + | + | 0/- | 0/- | 0/- | - | 0/- | - | - | 0/- | - |
| **fumaric acid** | - | - | - | - | + | - | - | - | - | - | - | - | - |
| **ethanol** | 0 | 0 | 0 | 0 | 0 | 0 | 0 | 0 | 0 | 0 | 0 | 0 | 0 |
| **glycerol** | 0 | 0 | 0 | 0 | 0/+ | 0 | 0/+ | 0 | 0 | 0 | 0 | 0 | 0 |
| **erythritol** | 0 | 0 | 0 | 0 | 0 | 0 | 0 | 0 | 0 | 0 | 0 | 0 | 0 |
| **galactitol** | 0 | 0 | 0/+ | 0/+ | + | 0 | 0 | 0 | 0 | 0 | 0 | 0 | 0 |
| **Gly** | + | + | + | + | + | + | + | ++ | + | 0 | 0 | + | + |
| **Leu** | + | + | ++ | +++ | + | 0 | + | ++ | + | 0 | 0 | + | 0 |
| **Val** | + | 0 | 0 | 0 | 0 | 0 | 0 | 0 | 0 | 0 | 0 | + | + |
| **Ile** | + | 0 | 0 | + | 0 | 0 | 0 | + | 0 | 0 | 0 | 0 | + |
| **Phe** | +++ | ++ | +++ | + | ++ | + | ++ | ++ | ++ | +++ | + | ++ | 0 |
| **Trp** | 0 | 0 | 0 | 0 | 0 | 0 | 0 | 0 | 0 | 0 | 0 | 0 | 0 |
| **Lys** | 0 | 0/- | + | + | 0 | 0 | 0 | 0 | 0 | 0 | 0 | 0 | 0 |
| **Ser** | + | + | + | + | + | + | 0 | + | 0 | 0 | 0 | + | + |
| **Thr** | 0 | 0 | + | ++ | + | 0 | 0 | + | 0 | 0 | 0 | + | + |
| **Cys** | 0 | 0 | 0 | 0 | 0 | 0 | 0 | 0/- | 0 | 0 | 0/- | 0 | 0 |
| **Hys** | 0 | 0 | 0 | 0 | 0 | 0 | 0 | 0 | 0 | 0 | 0 | 0 | 0 |
| **Ala** | ++ | + | ++ | +++ | ++ | + | ++ | +++ | ++ | + | + | ++ | 0 |
| **L-citrulline** | 0 | 0 | 0 | 0 | 0 | 0 | + | 0/- | 0 | 0 | + | 0 | 0 |
| **cis aconitate** | - | - | - | - | - | - | - | - | - | - | - | - | - |
| **inosine** | +++ | ++ | +++ | +++ | +++ | + | +++ | +++ | +++ | ++ | ++ | +++ | 0 |
| **Vanillin** | - | - | - | - | - | - | - | - | - | - | - | - | - |
| **orotic acid** | - | - | - | - | - | - | - | - | - | - | - | - | - |
| **gallic acid** | 0/- | 0/- | 0/- | 0 | 0 | 0/- | 0/- | 0/- | 0/- | 0/- | 0/- | 0/- | 0/- |
| **l-methylmannoside** | 0 | 0 | 0 | 0 | 0 | 0 | 0 | 0 | 0 | 0 | 0 | 0 | 0 |
| **Keto-isovalerianic acid** | 0 | 0 | 0 | 0 | + | 0 | 0 | 0 | 0 | 0 | 0 | 0 | + |
| **gentisine acid** | +++ | +++ | ++ | +++ | +++ | +++ | +++ | +++ | +++ | +++ | + | ++ | ++ |
| **piruvate** | + | 0 | 0 | ++ | ++ | 0 | + | + | + | 0 | + | + | +++ |
| **Met** | 0 | 0 | 0 | 0 | 0 | 0 | 0 | 0 | 0 | 0 | 0 | 0 | 0 |
| **Arg** | ++ | + | + | ++ | + | ++ | ++ | ++ | ++ | ++ | ++ | ++ | 0 |
| **Tyr** | ++++ | +++ | +++ | ++ | +++ | +++ | +++ | +++ | ++++ | +++ | +++ | ++++ | ++ |
| **ascorbic acid** | 0/- | 0/- | 0/- | 0/- | 0 | 0/- | 0/- | 0/- | 0/- | 0/- | 0/- | 0/- | - |
| **protocatechuic acid** | 0/- | 0/- | 0/- | 0 | 0 | 0 | 0 | 0/- | 0/- | 0/- | 0/- | 0/- | 0/- |
| **α-methyl-D-xyloside** | +++ | ++ | ++ | ++ | ++ | ++ | ++ | + | 0 | 0 | 0 | ++ | ++ |
| **melibiose** | ++ | ++ | ++ | +++ | ++ | ++ | + | ++ | ++ | ++ | ++ | ++ | +++ |
| **Glycerol-l-monoacetate** | +++ | ++ | ++ | +++ | +++ | ++ | +++ | +++ | ++ | ++ | +++ | +++ | +++ |
| **mannite** | +++ | ++ | +++ | +++ | +++ | ++ | +++ | +++ | +++ | +++ | +++ | ++++ | 0 |
