# Supplementary material for: Lichtheimia Species Exhibit Differences in Virulence Potential
Source: PLoS One. 2012 Jul 20;7(7):e40908. doi: 10.1371/journal.pone.0040908 (PMC3401187; doi:10.1371/journal.pone.0040908)
Supplement: Table S3 — Utilization of different nitrogen sources by 12 representative strains of Lichtheimia species. No Growth is indicated as ‘0’, inhibition as ‘-’ and growth as ‘+’. (DOCX) [file pone.0040908.s004.docx]

|  |  | Potassium nitrate | Urea | Glycine | Asparagine | Tryptophane | | Casamino acids | Peptone | BSA | Gelatine |
| --- | --- | --- | --- | --- | --- | --- | --- | --- | --- | --- | --- |
| *L. corymbifera* | FSU 9682 | ++ | + | ++ | ++ | + | ++ | | ++ | ++ | ++ |
|  | FSU 10164 | ++ | 0/+ | ++ | ++ | - | ++ | | ++ | ++ | ++ |
| *L. ramosa* | FSU 6197 | ++ | ++ | ++ | ++ | 0/+ | ++ | | ++ | ++ | ++ |
|  | FSU 9927 | ++ | 0/+ | 0/+ | ++ | - | ++ | | ++ | ++ | ++ |
|  | FSU 10166 | ++ | ++ | ++ | ++ | 0/+ | ++ | | ++ | ++ | ++ |
| *L. ornata* | FSU 10165 | ++ | 0/+ | 0/+ | ++ | - | ++ | | ++ | 0/+ | ++ |
|  | FSU 10167 | ++ | + | ++ | ++ | + | ++ | | ++ | + | ++ |
| *L. hyalospora* | FSU 10160 | ++ | - | ++ | ++ | - | ++ | | ++ | ++ | + |
|  | FSU 10161 | ++ | 0/+ | ++ | ++ | 0/+ | ++ | | 0/+ | + | ++ |
|  | FSU 10162 | ++ | 0/+ | ++ | ++ | + | ++ | | ++ | ++ | ++ |
|  | FSU 10163 | ++ | 0/- | + | + | - | ++ | | + | + | + |
| *L. sphaerocystis* | FSU 10079 | ++ | 0/- | + | + | - | ++ | | + | + | ++ |

**Table S3. Utilization of different nitrogen sources by 12 representative strains of *Lichtheimia* species.** No Growth is indicated as '0', inhibition as '-' and growth as '+'.
